# Targeting Rev-Erbα to protect against ischemia-reperfusion-induced acute lung injury in rats

**DOI:** 10.1186/s12931-023-02547-7

**Published:** 2023-10-12

**Authors:** Shi-Jye Chu, Wen-I Liao, Hsin-Ping Pao, Shu-Yu Wu, Shih-En Tang

**Affiliations:** 1 Division of Rheumatology, Immunology, and Allergy, Department of Internal Medicine, Tri- Service General Hospital, National Defense Medical Center, Taipei, Taiwan; 2 Department of Emergency Medicine, Tri-Service General Hospital, National Defense Medical Center, Taipei, Taiwan; 3 Institute of Aerospace and Undersea Medicine, National Defense Medical Center, Taipei, Taiwan; 4 Division of Pulmonary and Critical Care Medicine, Department of Internal Medicine, Tri- Service General Hospital, National Defense Medical Center, No. 325, Section 2, Chenggong Road, Neihu, Taipei114, Taiwan

**Keywords:** Acute lung injury, Ischemia-reperfusion, Circadian clocks, Rev-Erbα, SR9009, SR8278

## Abstract

**Background:**

The dysregulation of local circadian clock has been implicated in the pathogenesis of a broad spectrum of diseases. However, the pathophysiological role of intrinsic circadian clocks Rev-Erbα in ischemia-reperfusion (IR)-induced acute lung injury (ALI) remains unclear.

**Methods:**

The IR-ALI model was established by subjecting isolated perfused rat lungs to 40 min of ischemia followed by 60 min of reperfusion. Rats were randomly assigned to one of six groups: control, control + SR9009 (Rev-Erbα agonist, 50 mg/kg), IR, and IR + SR9009 at one of three dosages (12.5, 25, 50 mg/kg). Bronchoalveolar lavage fluids (BALF) and lung tissues were obtained and analyzed. In vitro experiments utilized mouse lung epithelial cells (MLE-12) exposed to hypoxia-reoxygenation (HR) and pretreated with SR9009 (10 µM/L) and Rev-Erbα siRNA.

**Results:**

SR9009 exhibited a dose-dependent reduction in lung edema in IR-ALI. It significantly inhibited the production of TNF-α, IL-6, and CINC-1 in BALF. Moreover, SR9009 treatment restored suppressed IκB-α levels and reduced nuclear NF-κB p65 levels in lung tissues. In addition, a SR9009 mitigated IR-induced apoptosis and mitogen-activated protein kinase (MAPK) activation in injured lung tissue. Finally, treatment with Rev-Erbα antagonist SR8278 abolished the protective action of SR9009. In vitro analyses showed that SR9009 attenuated NF-κB activation and KC/CXCL-1 levels in MLE-12 cells exposed to HR, and these effects were significantly abrogated by Rev-Erbα siRNA.

**Conclusions:**

The findings suggest that SR9009 exerts protective effects against IR-ALI in a Rev-Erbα-dependent manner. SR9009 may provide a novel adjuvant therapeutic approach for IR-ALI.

**Supplementary Information:**

The online version contains supplementary material available at 10.1186/s12931-023-02547-7.

## Introduction

Ischemia-reperfusion-induced acute lung injury (IR-ALI) represents a complex condition characterized by the interruption and subsequent restoration of blood supply to the lungs, leading to aberrant physiological processes that disrupt vascular permeability and incite dysregulated inflammatory responses [1–3]. The pathogenesis of IR-ALI involves the excessive generation of reactive oxygen species (ROS), upregulation of pro-inflammatory cytokines, activation of the nuclear factor-κB (NF-κB) pathway, infiltration of neutrophils into the alveoli, and dysfunction of both the epithelium and endothelium. Additionally, lung cell apoptosis plays a significant role in the development of pulmonary dysfunction associated with IR injury. These pathophysiological events contribute to heightened pulmonary vascular resistance, augmented microvascular permeability, and the onset of lung edema. IR-ALI can arise in various clinical scenarios, encompassing lung transplantation, major surgical procedures, cardiopulmonary bypass surgery, pulmonary embolism, resuscitation for cardiac arrest, and hemorrhagic shock [1–3]. Despite advancements in therapeutic strategies, the mortality rate of ALI and its severe form, acute respiratory distress syndrome (ARDS), remains alarmingly high, ranging from 30 to 40% [1–3]. Presently, there is a notable dearth of clinically effective medications specifically tailored for the treatment of IR-ALI, with available interventions primarily focused on supportive care. Therefore, the identification and development of novel therapeutic approaches to address IR-ALI constitute an imperative research endeavor.

Rev-Erbα, a nuclear receptor also known as Nr1d1, stands as a pivotal component of the circadian clock machinery, exerting transcriptional repression as its primary function. Recent studies underscore the profound influence of the circadian clock system on vital physiological processes, including energy metabolism, oxidative stress, inflammation, cellular proliferation, and senescence [4, 5]. Consequently, Rev-Erbα emerges as an enticing therapeutic target amenable to modulation via available small-molecule agonists and antagonists [4]. A growing body of evidence substantiates the detrimental effects of clock disturbances on pulmonary function and the development of lung disorders [5, 6]. Various factors, ranging from air pollutants, particulate matter, xenobiotic detoxification, cigarette smoke, shift work, jet lag, hypoxia/hyperoxia, ventilator-induced ALI, to bacterial and viral pathogens, can disrupt the molecular clock within the lungs, thereby exacerbating the progression of lung diseases [6]. These findings emphasize the role of clock disruption in orchestrating diverse cellular and molecular changes during lung disease development. Moreover, recent investigations shed light on the regulatory role of Rev-Erbα in the context of lung injury, pulmonary fibrosis, and chronic obstructive pulmonary disease (COPD), where circadian clock synchronization appears to be of paramount importance.

Animal models with disrupted or inhibited expression of Rev-Erbα display adverse effects on pulmonary function, increased vulnerability to infection, and an aggravated inflammatory response, resulting in unfavorable recovery outcomes. Conversely, animals exhibiting Rev-Erbα overexpression or administered a Rev-Erbα agonist exhibit diminished production of inflammatory cytokines and improved lung damage [7–9]. Despite these observations implying the significance of Rev-Erbα in lung inflammation, the precise contribution of Rev-Erbα to IR-ALI remains inadequately comprehended. In this study, we postulate that the diminution of Rev-Erbα abundance induced by IR potentiates both pulmonary and cellular inflammatory responses. Additionally, we hypothesize that the activation of Rev-Erbα using small molecule agonists, such as SR9009, would mitigate IR-ALI.

## Methods

### Protocol for preparing isolation and perfusion of rat lungs

In adherence to the ethical guidelines set forth in the Guide for the Care and Use of Laboratory Animals (National Institutes of Health, National Academy Press 1996), meticulous measures were implemented to uphold the welfare of the rats involved in this research. The experimental design obtained approval from the Animal Review Committee of the National Defense Medical Center, Taipei, Taiwan (Permit Number: IACUC-20-216). For the establishment of isolated perfused rat lungs, male Sprague-Dawley rats weighing 350 ± 20 g were subjected to ventilation using a gas mixture composed of 21% O_2_ and 5% CO_2_ at a rate of 60 breaths per minute. The tidal volume was set at 3 ml, while a positive end-expiratory pressure of 1 cm H_2_O was maintained, as previously described [10]. Heparin, at a dosage of 1 U per gram of body weight (BW), was administered via injection into the right ventricle, accompanied by the withdrawal of 10 cc of blood from the same location. Cannulation of the pulmonary artery and left ventricle was performed using silicon tubing, enabling the perfusion of the lungs with a physiological salt solution supplemented with 4% bovine serum albumin. To create a “half-blood” solution, the collected rat blood (10 cc) was mixed with the perfusate prior to recirculation. Throughout the experiment, the isolated lung was placed on an electronic balance to enable the continuous monitoring of real-time changes in lung weight (LW). Additionally, both the pulmonary venous pressure (PVP) and pulmonary arterial pressure (PAP) were constantly recorded using pressure transducers connected to the side arm of the cannula, following established techniques [11].

### Assessing capillary permeability

In accordance with previously established methods, we sought to ascertain the capillary permeability (K_f_) by evaluating the augmentation in lung weight induced by an elevation in venous pressure. The quantification of K_f_ was subsequently expressed in milliliters per minute per centimeter water per 100 g of lung tissue, following the established protocol [12, 13].

### Animal experimental design

The present study employed a randomized experimental design in which rats were assigned to one of eight groups. These included a control group that received dimethyl sulfoxide (DMSO) (n = 6), a drug control group that received 50 mg/kg of SR9009 (50 mg/kg BW, a Rev-Erbα agonist, intraperitoneal injection (i.p.), Sigma-Aldrich, USA), a group subjected to lung IR (IR, n = 6), and three groups that received different doses of SR9009 (12.5 mg, 25 mg, or 50 mg/kg BW) along with lung IR (n = 6 per group), IR + SR8278 (5 mg/kg BW, a Rev-Erbα antagonist), or IR + SR8278 (5 mg/kg BW) + SR9009 (50 mg/kg BW) group. To facilitate intraperitoneal injection, SR9009 was dissolved in 0.5% DMSO and administered 60 min before inducing lung IR. In the IR + SR8278 + SR9009 group, rats were pretreated with SR8278 for 30 min prior to SR9009 injection. The dosages of SR9009 and SR8278 used in this study were based on previous research and preliminary investigations [7, 9] (Supplementary Fig. 1). Lung IR was induced by stopping ventilation and perfusion, leading to ischemia, and then maintaining the lungs in a deflated state for 40 min, followed by 60 min of ventilation and perfusion. In contrast, the control group received perfusate alone for 100 min.

### Evaluating the ratio of water present in the lung tissue

Following the experiment, the right lung was surgically removed at the hilar region. The wet weight of the lung was measured, and the lung weight-to-body weight (LW/BW) ratio was calculated to evaluate the degree of lung edema. Furthermore, a portion of the right upper lung lobe was excised and weighed, and then dried for 48 h at 60 °C to obtain its dry weight. Subsequently, the wet-to-dry (W/D) weight ratio was computed.

### Examining protein, cytokine, and cell levels in bronchoalveolar lavage fluid (BALF)

Following the experiment, the left lung underwent two rounds of lavage using 2.5 ml of saline to obtain BALF. This fluid was subsequently subjected to centrifugation at 200 × g for 10 min at room temperature. The protein content of the BALF was assessed using a bicinchoninic acid protein assay kit (Pierce, Rockford, IL, USA). Commercial rat ELISA kits (R&D Systems Inc., Minneapolis, MN, USA) were employed to measure BALF levels of tumor necrosis factor-α (TNF-α), cytokine-induced neutrophil chemoattractant-1 (CINC-1), and interleukin-6 (IL-6). The total cell count in the BALF was determined utilizing a previously described method [11, 13].

### Quantification of malondialdehyde (MDA), H_2_O_2_, and glutathione levels in lung tissue

Malondialdehyde (MDA) levels were assessed using an established protocol [13]. The measurement involved determining the reaction product of MDA with thiobarbituric acid reactive substances at an absorbance of 532 nm. The resulting values were expressed in nmol/mg protein. To measure the levels of glutathione and hydrogen peroxide (H_2_O_2_) in the lung tissues, a fluorometric glutathione detection assay kit and a Hydrogen Peroxide Assay Kit from Abcam (Cambridge, MA, USA) were utilized.

### Western blot analysis

Lung tissue homogenate and cell protein lysates, with a protein amount of 30 µg per lane, were subjected to separation using sodium dodecyl sulfate-polyacrylamide gel electrophoresis (SDS-PAGE) on a 10–12% gel as described in a previous study [13]. Following separation, the proteins were transferred onto polyvinylidene fluoride membranes, following the established protocol. To investigate the presence of specific proteins, the membranes were probed with primary antibodies against β-actin, Rev-Erbα, Bcl-2, NF-κB p65, phospho-NF-κB p65, IκB-α, extracellular signal-regulated kinase( ERK)1/2, phospho-ERK1/2, c-Jun N-terminal kinase (JNK), phospho-JNK, p38, phospho-p38, Protein kinase B (Akt), and phospho-Akt. The obtained results are presented as the relative ratio of the target protein to the reference protein, allowing for the quantification and comparison of protein levels within the samples.

### Immunohistochemistry

In accordance with previously published methods, the immunohistochemical staining protocol for myeloperoxidase (MPO) was employed [14]. Lung tissue sections, measuring 4 mm in thickness, were obtained from paraffin blocks. To eliminate peroxidase activity, the sections were subjected to dewaxing and treated with 3% H_2_O_2_ and 100% methanol for a duration of 15 min. For MPO staining, a primary anti-MPO rabbit antibody (diluted at 1:100, obtained from Thermo Fisher Scientific, Rockford, IL, USA) was applied to the sections, followed by three washes with phosphate-buffered saline (PBS) for 5 min each. Subsequently, a rat-specific horseradish peroxidase polymer anti-rabbit antibody (manufactured by Nichirei Corporation, Tokyo, Japan) was added and allowed to incubate for 30 min. The sections were then washed thrice with PBS. To visualize the MPO staining, a horseradish peroxidase substrate was applied for a duration of 3 min. Hematoxylin was used as a counterstain for the sections. All staining tests were performed in triplicate to ensure consistency and reliability of the results.

### Histopathological evaluation using microscopy

To examine the tissue architecture and detect any pathological alterations, hematoxylin and eosin staining were applied on lung tissue sections that were embedded in paraffin. The amount of polymorphonuclear neutrophils present in the interstitium was measured by counting cells in 10 high-power field views (×400) chosen randomly and then averaging them. This was done by two laboratory technicians who were unaware of the slide’s source. Furthermore, a semi-quantitative grading was carried out on the hematoxylin and eosin sections to evaluate the extent of pathological lesions, as previously described [15] .

### A TUNEL assay to analyze DNA fragmentation in the lung tissue

The TUNEL assay was performed on 5 mm-thick paraffin-embedded lung tissue sections using the FragELTM DNA Fragmentation Detection Kit and Fluorescent-TdT Enzyme (Merck Millipore, Darmstadt, Germany) according to the manufacturer’s instructions. TUNEL-positive nuclei were detected through fluorescence microscopy.

### Induction of hypoxia-reoxygenation (HR) in mouse lung epithelial (MLE)-12 cells

The MLE-12 cells were cultured in DMEM/F-12 medium, which was supplemented with 10% fetal bovine serum, penicillin (100 U/mL), and streptomycin (10 mg/mL). The cells were maintained at a temperature of 37 °C and a CO_2_ concentration of 5% in a humidified air environment, following previously established protocols [12, 16]. To induce hypoxia-reoxygenation (HR), the MLE-12 cells were subjected to 2 h of hypoxia (1% O_2_, 5% CO_2_, and 94% N_2_) at 37 °C, followed by 1 h of reoxygenation (5% CO_2_ and 95% room air). Prior to HR induction, the cells were treated with different substances: either a control vehicle, SR9009 (10 µM), or Rev-Erbα siRNA (25 nM). The dosage of SR9009 and Rev-Erbα siRNA was determined through reference to a prior study [17, 18] and analysis of Western blot results (Supplementary Fig. 2). Transfection of Rev-Erbα siRNA was accomplished by incubating the cells with DharmaFECT™ 1 siRNA transfection reagent (Dharmacon Inc. Chicago, IL, USA) for 24 h. As negative controls, MLE-12 cells treated with a non-targeting control siRNA were used. After 24 h, the culture medium was replaced with the appropriate medium. At 48 h from the initiation of Rev-Erbα siRNA treatment, the MLE-12 cells were exposed to 2 h of hypoxia followed by 1 h of reoxygenation. Protein extraction was conducted by lysing the cells, and Western blot analysis was performed. Additionally, the supernatant was collected and subjected to keratinocyte chemoattractant (KC) analysis using a mouse KC/CXCL1 ELISA kit (R&D, Inc.). All experiments were conducted in triplicate.

### Data analysis

The data obtained from the experiments were subjected to statistical analysis using GraphPad Prism 5 software (GraphPad Software, San Diego, CA, USA). The results are expressed as means accompanied by standard deviations. To assess the differences between the groups, a one-way analysis of variance (ANOVA) was performed, followed by *post-hoc* Bonferroni tests for conducting multiple comparisons. Lung weight gain and pulmonary artery pressure (PAP) were compared among the groups using a repeated measures two-way ANOVA, followed by *post-hoc* Bonferroni tests. The threshold for determining statistical significance was set at a *p*-value of less than 0.05.

## Results

### SR9009 attenuated IR-induced lung edema

**Fig. 1:**
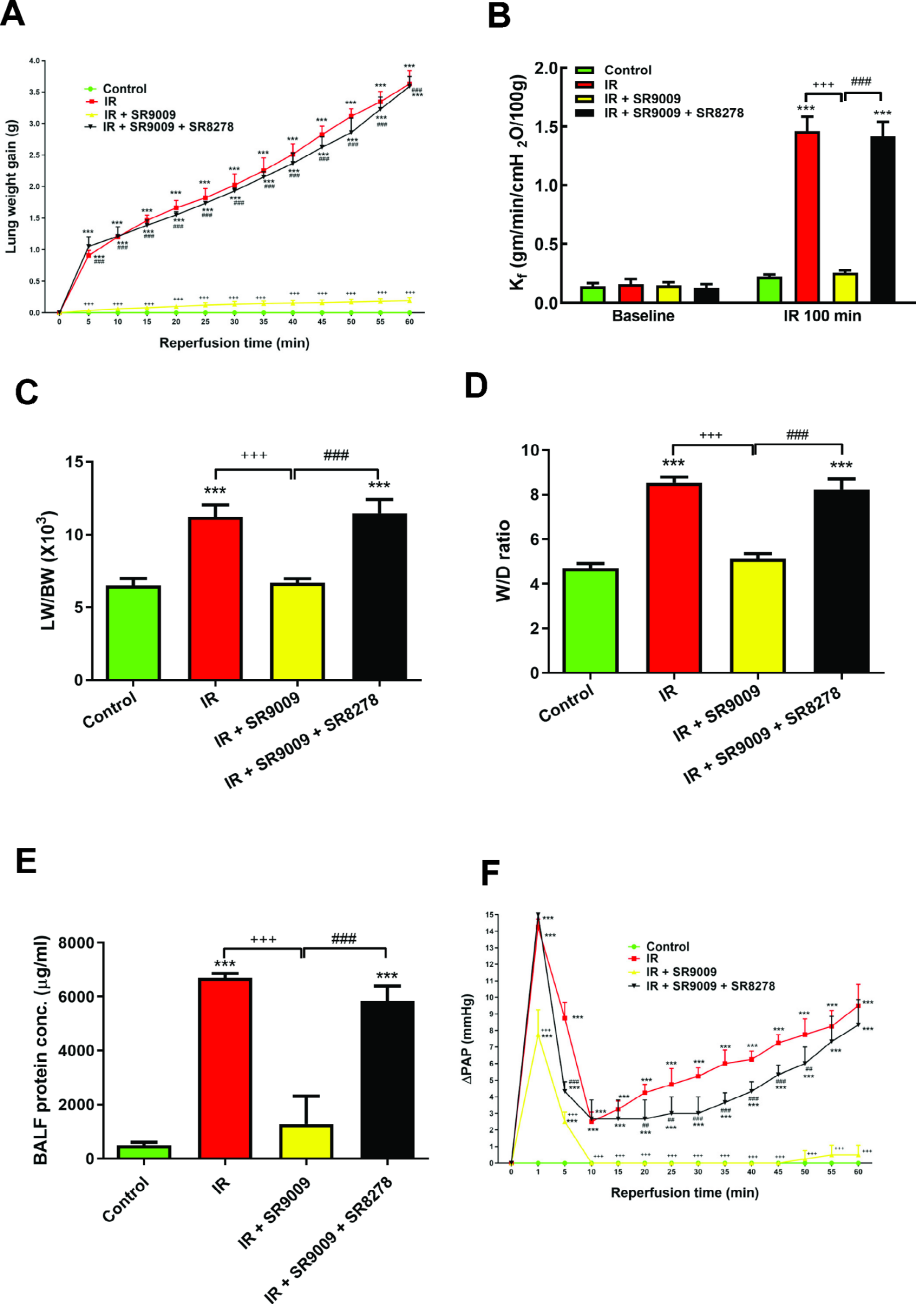
Effect of SR9009 on pulmonary edema. Lung weight gain (**A**), K_f_ (**B**), lung weight/body weight (LW/BW) ratios (**C**), lung wet/dry (W/D) weight ratios (**D**), and protein concentrations in the bronchoalveolar lavage fluid (BALF) (**E**) and pulmonary artery pressure (**F**) significantly increased in the ischemia-reperfusion (IR) group. Treatment with SR9009 significantly attenuated the increase in these parameters. The protective effect of SR9009 was abrogated by the addition of SR8278. Data are expressed as means ± SD (*n* = 6 per group). ^***^p < 0.001 compared to control group; ^+++^*p* < 0.001 compared to IR group; ^##^*p* < 0.01, ^###^*p* < 0.001 compared to IR + SR9009 50 mg group

In comparison to the control group treated with a vehicle, the induction of IR injury resulted in a notable increase in lung weight gain, vascular filtration coefficient (K_f_), ratio of lung weight to body weight (LW/BW), ratio of wet to dry lung weight (W/D weight), and protein concentrations in the BALF. In addition, the administration of SR8278 exhibited a trend towards exacerbating IR damage, although this trend did not reach statistical significance (not shown). Conversely, rats that received pretreatment with SR9009 showed a significant reduction in IR-induced lung edema in a dose-dependent manner, as demonstrated in Supplementary Fig. 1A-F. Notably, the protective effects of SR9009 on IR-induced lung edema were significantly diminished when the SR8278 was added, as shown in Fig. 1A-E.

### SR9009 reduces pulmonary artery pressure in IR-induced lung injury

Upon induction of IR, there was an initial rapid rise in pulmonary artery pressure (PAP), which later declined after reperfusion, as indicated in Fig. 1F. Even after 60 min of reperfusion, the PAP in the IR-exposed lungs remained significantly higher compared to the baseline and the control group, where PAP remained stable during the 100-minute observation period. Administration of SR9009 effectively mitigated the IR-induced changes in PAP, displaying a significant suppression of PAP alterations (*p* < 0.05; Fig. 1F). However, the addition of SR8278 significantly counteracted the protective effects of SR9009 on preventing the elevation of PAP.

### SR9009 attenuated the level of proinflammatory cytokines and total cell counts in the BALF

**Fig. 2:**
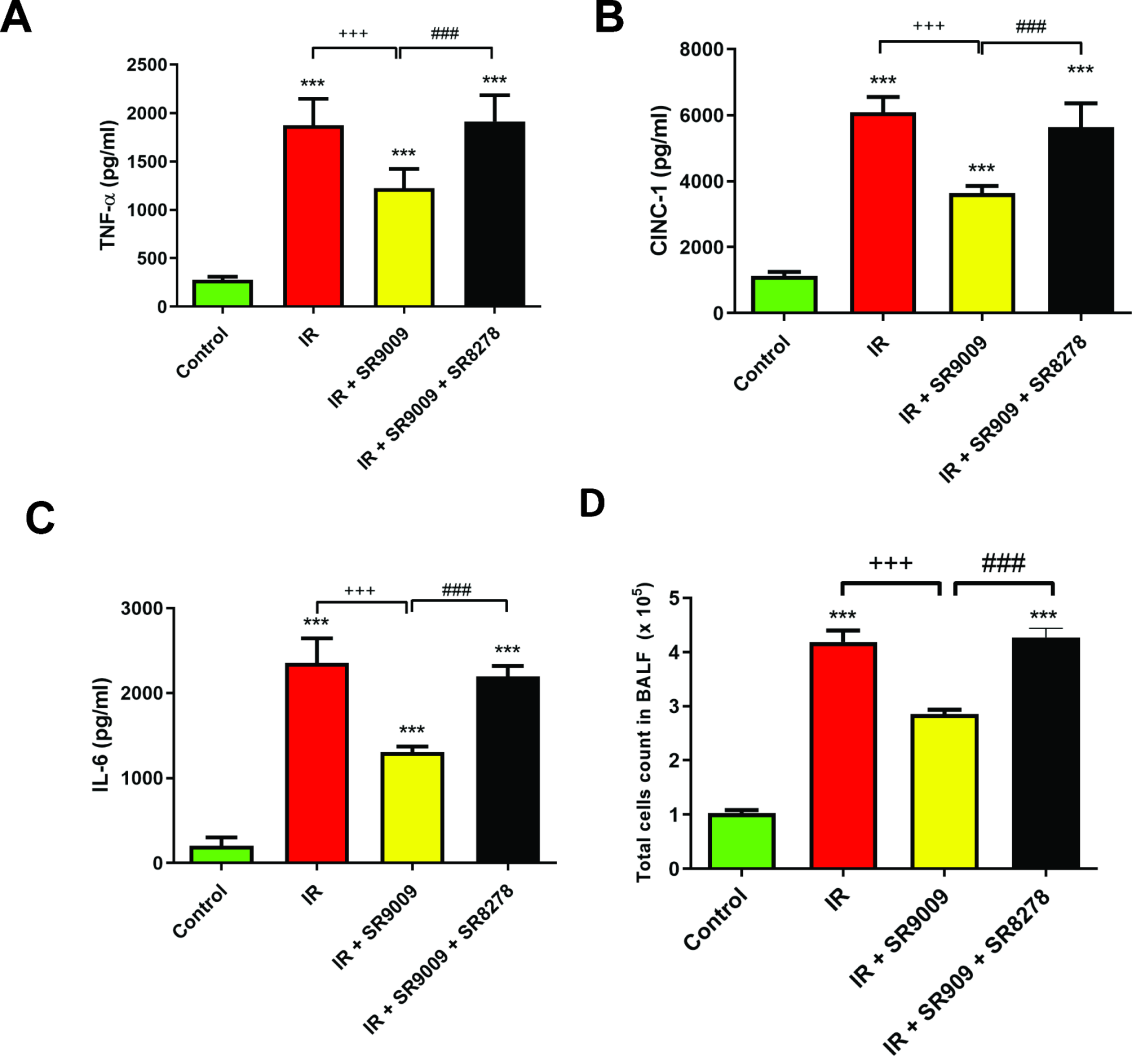
Effect of SR9009 on TNF-α, CINC-1 and IL-6 levels, and total cell counts in bronchoalveolar lavage fluid (BALF). The levels of TNF-α **(A)**, CINC-1 **(B)**, and IL-6 **(C)**, as well as the total cell counts **(D)** in the bronchoalveolar lavage fluid (BALF), exhibited a significant increase in the ischemia-reperfusion (IR) group. However, the administration of SR9009 effectively reduced these elevations in the BALF. Notably, the protective effect of SR9009 was blocked by the addition of SR8278. Data are expressed as mean ± SD (n = 6 per group). ^***^p < 0.001 compared with the control group; ^+++^*p* < 0.001 compared with the IR group; ^##^*p* < 0.01, ^###^*p* < 0.001 compared to IR + SR9009 50 mg group

The levels of TNF-α, CINC-1, IL-6, and total cell counts in BALF showed a significant increase in the IR group compared to the control group (*p* < 0.05; Fig. 2). In contrast, rats pretreated with SR9009 exhibited a significant reduction in TNF-α, CINC-1, IL-6 levels, and total cell count in BALF induced by IR injury, when compared to rats subjected to IR injury alone (Fig. 2). However, the protective effects of SR9009 on the IR-induced increase were significantly blocked by the addition of SR8278 (Fig. 2).

### SR9009 increased Rev-Erbα protein expression in IR lung tissue

**Fig. 3:**
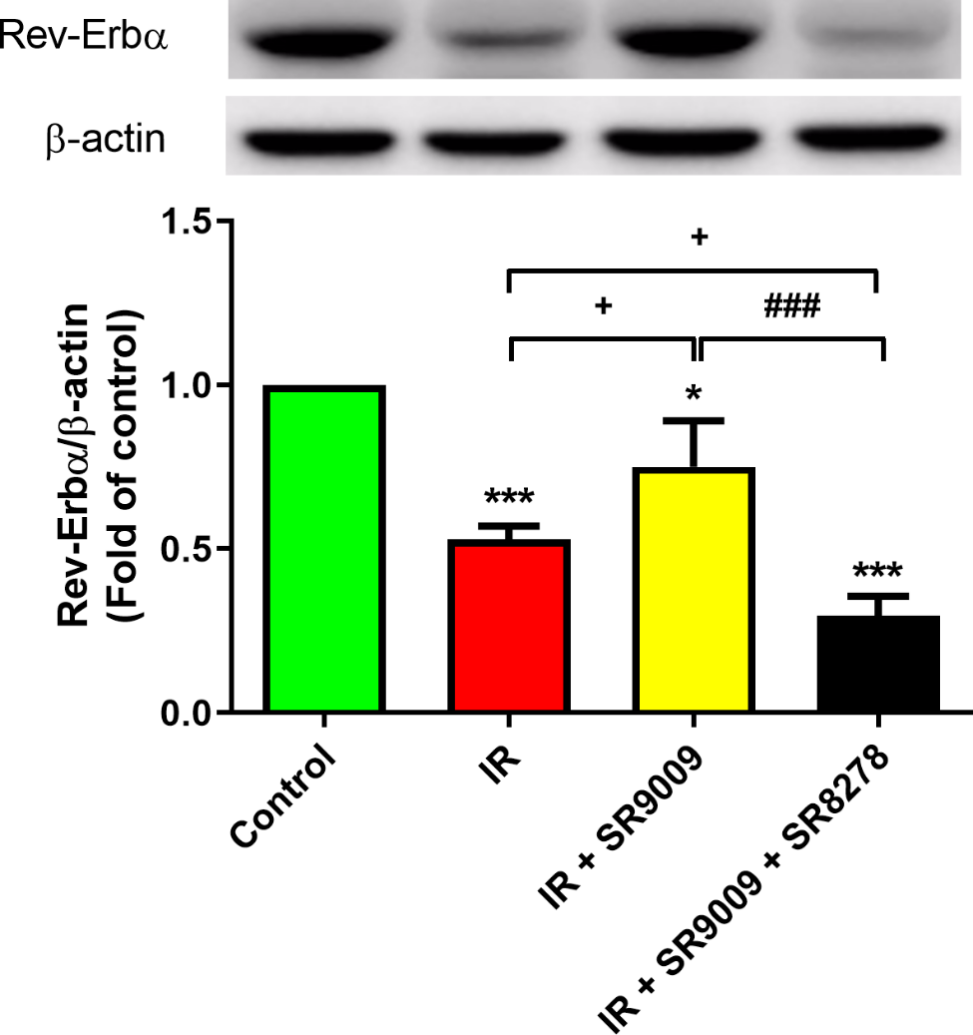
Effect of SR9009 on levels of Rev-Erbα proteins in the lung tissue. The expression levels of Rev-Erbα proteins in the lung of each group were detected by Western blot. The expression of Rev-Erbα protein was diminished by the process of ischemia-reperfusion (IR), but administration of SR9009 substantially augmented the expression of Rev-Erbα protein when compared to the IR group. However, the presence of SR8278 significantly impeded the effects of SR9009 on the IR-induced increase in Rev-Erbα expression. β-actin is used as loading controls for cytoplasmic proteins. Representative blots are shown. Data are expressed as mean ± SD (n = 3 per group). ^*^p < 0.05, ***p < 0.001, compared to control group; ^+^*p* < 0.05, compared to IR group; ^###^*p* < 0.01, compared to IR + SR9009 50 mg group

The Western blot analysis revealed a notable decrease in the expression levels of Rev-Erbα proteins in the lung tissue of the IR group when compared to the control groups (*p* < 0.05; Fig. 3). However, treatment with SR9009 significantly increased the expression of Rev-Erbα protein compared to the IR group (*p* < 0.05; Fig. 3). Notably, the protective effects of SR9009 on the IR-induced increase in Rev-Erbα protein expression were significantly blocked by the addition of SR8278.

### SR9009 decreases MDA and H_2_O_2_ levels, reduces MPO-positive cells, and increases glutathione level in IR lung tissue

**Fig. 4:**
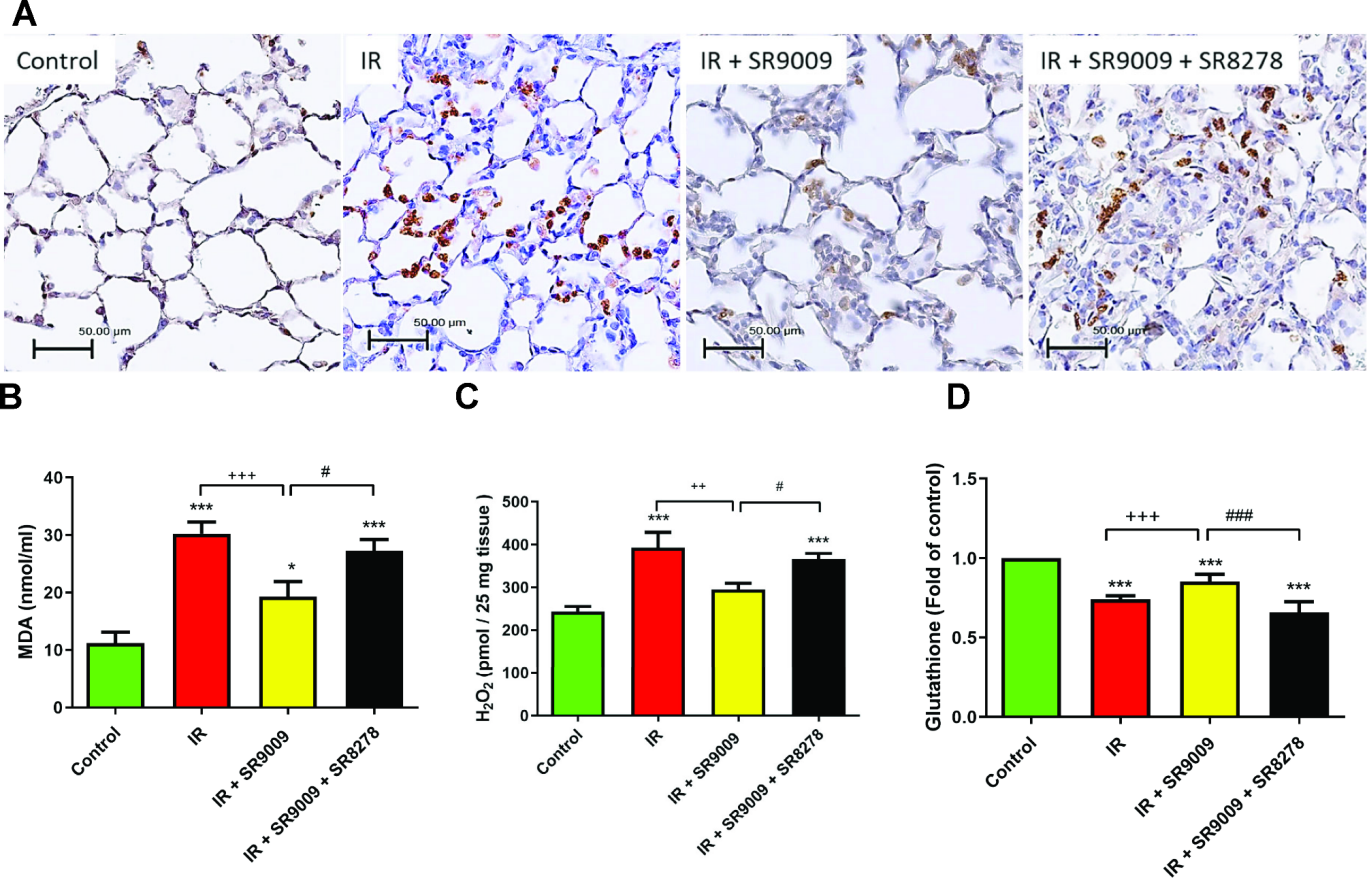
Effect of SR9009 on oxidative stress in the lung tissue. Ischemia-reperfusion (IR) triggered a notable increase in the number of MPO-positive cells and elevated levels of MDA and H_2_O_2_, while also reducing the glutathione level in lung tissue. In contrast, the use of SR9009 significantly reduced these effects caused by IR. However, the introduction of SR8278 counteracted the protective effects of SR9009.Data are expressed as mean ± SD (n = 6 per group). ^*^p < 0.05, ***p < 0.001, compared to control group; ^++^*p* < 0.01,^+++^*p* < 0.001, compared to IR group; ^#^*p* < 0.05, ^###^*p* < 0.001, compared to IR + SR9009 50 mg group

Compared to the control group, the IR group exhibited a significant rise in the number of MPO-positive cells, as well as increased levels of MDA and H_2_O_2_, along with a reduction in glutathione level in lung tissue (*p* < 0.05; Fig. 4A–D). Conversely, the administration of SR9009 significantly attenuated these IR-induced effects. However, the addition of SR8278 blocked the protective effects of SR9009.

### SR9009 attenuated neutrophil infiltration and histological changes in IR lung tissue

**Fig. 5:**
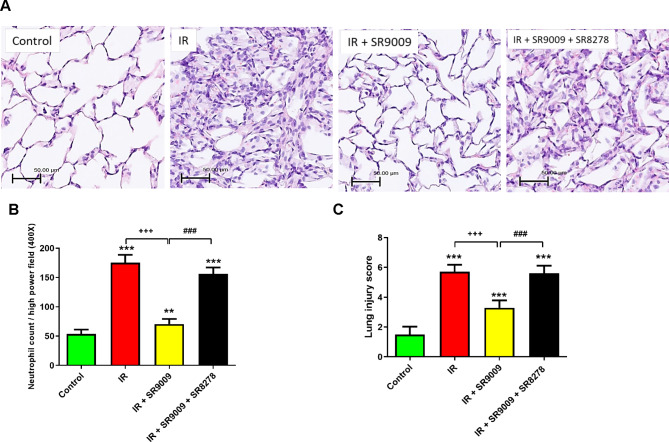
Effect of SR9009 on lung pathology. As shown by a representative micrograph of lung tissue (400 × magnification) **(A)**, neutrophil infiltration and septal edema increased in the ischemia-reperfusion (IR) group. SR9009 treatment significantly attenuated these histopathological changes, the numbers of neutrophils per high power field (400 × magnification) **(B)**, and the lung injury scores **(C)**. The protective effect of SR9009 was abrogated by the addition of SR8278. Scale bar: 50 μm. Data are expressed as mean ± SD (n = 6 per group). ^**^p < 0.01, ***p < 0.001, compared to control group; ^+++^*p* < 0.001, compared to IR group; ^###^*p* < 0.001, compared to IR + SR9009 50 mg group

The histological assessment exhibited a significant increase in interstitial thickening and cellular infiltration in the IR group in comparison to the control group (Fig. 5A). Nonetheless, the administration of SR9009 substantially decreased these histological changes, neutrophil infiltration (Fig. 5B), and lung injury scores (Fig. 5C) in the IR group. Moreover, the addition of the SR8278 significantly blocked the effect of SR9009.

### SR9009 inhibited apoptosis in IR lung tissue

**Fig. 6:**
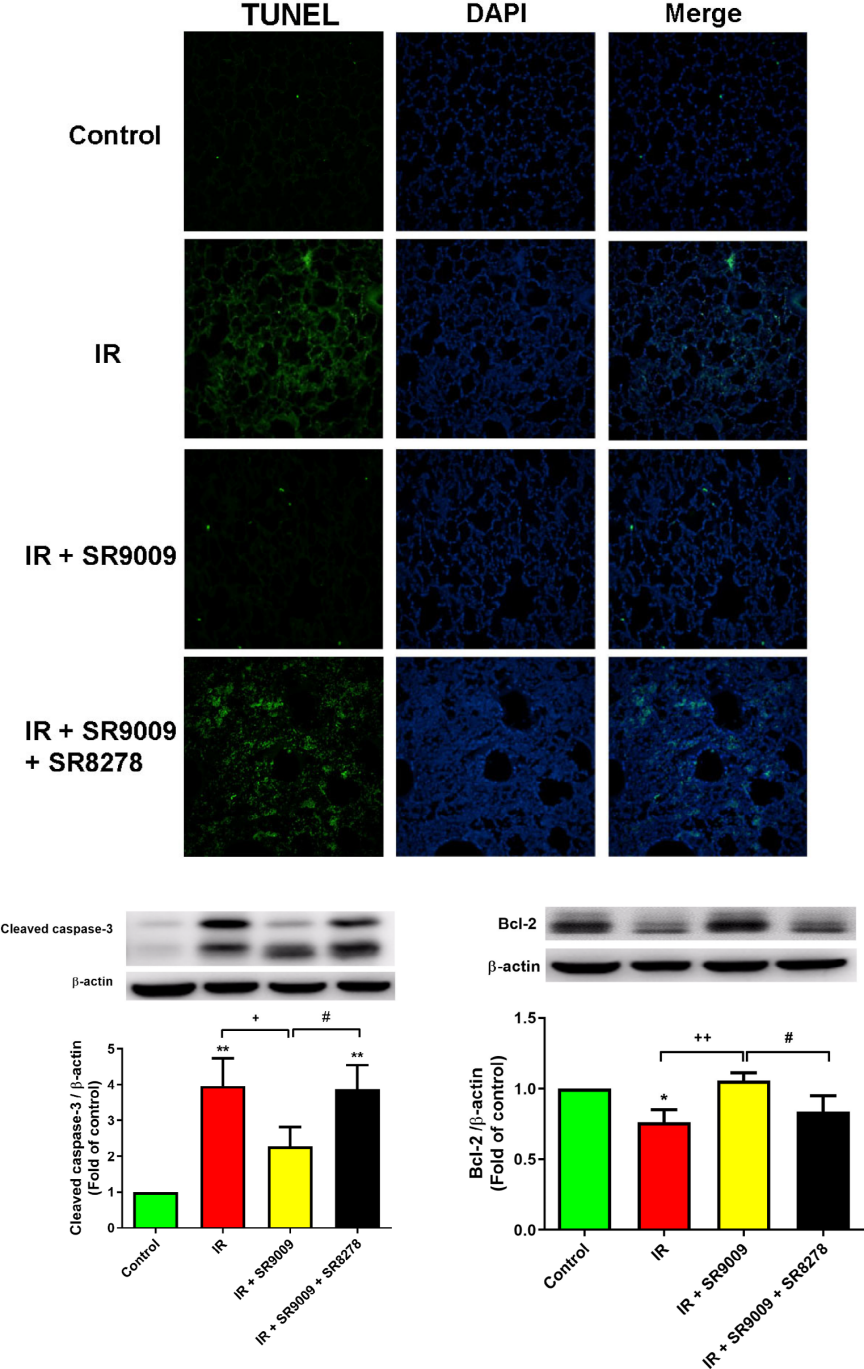
Effect of SR9009 on apoptosis in lung tissue. **(A)** Lung tissue was subjected to TUNEL assay. **(B-C)** Protein expressions of cleaved caspase-3 **(B)** and Bcl-2 **(C)** in lung tissues were analyzed using Western blot. The cytoplasmic proteins were normalized using β-actin as a loading control. Representative blots are presented. Data are expressed as mean ± SD (n = 3 per group). *p < 0.05, ^**^p < 0.01, compared to control group; ^+^*p* < 0.05, ^++^*p* < 0.01, compared to IR group; ^#^*p* < 0.05, compared to IR + SR9009 50 mg group

The number of TUNEL-positive cells (Fig. 6A) and the protein levels of cleaved caspase-3 (Fig. 6B) were significantly higher, and the protein level of Bcl-2 was significantly lower (Fig. 6C) in the lungs of the IR group compared to the control group. But SR9009 treatment significantly attenuated the severity of these apoptosis-related changes in lungs exposed to IR. However, the addition of the SR8278 significantly blocked the effect of SR9009.

### SR9009 inhibited the NF-κB signaling pathway in IR lung tissue

**Fig. 7:**
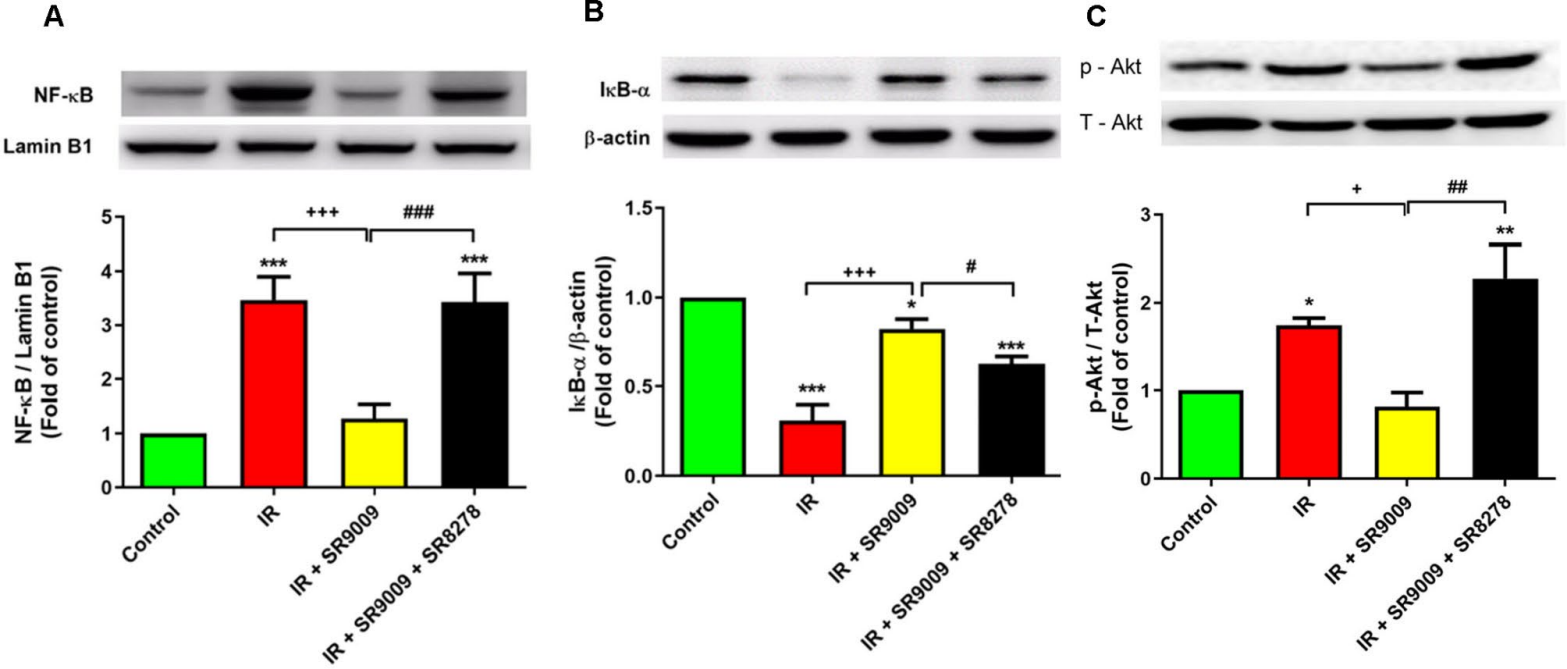
Effect of SR9009 on NF*-*κB activation in lung tissues. The administration of SR9009 in ischemia-reperfusion (IR)-induced lung injury resulted in reduced levels of nuclear NF-κB p65 **(A)** and Akt phosphorylation **(C)**, while increasing the levels of IκB-α **(B)**. However, the protective effect of SR9009 was negated by the addition of SR8278. The protein markers Lamin B1 and β-actin were utilized as loading controls for nuclear and cytoplasmic proteins, respectively. Representative Western blots are displayed. Data are expressed as mean ± SD (n = 3 per group). *p < 0.05, ^***^p < 0.001, compared to control group; ^+^*p* < 0.05, ^+++^*p* < 0.001, compared to IR group; ^#^*p* < 0.05, ^##^*p* < 0.01, ^###^*p* < 0.001, compared to IR + SR9009 50 mg group

Figure 7 A and C show a significant increase in the levels of NF-κB p65 in the nucleolus and Akt phosphorylation in the IR group. Conversely, Fig. 7B demonstrates a significant decrease in the cytoplasmic level of IκB-α compared to the control group. Treatment with SR9009 resulted in a significant rise in IκB-α levels while reducing NF-κB p65 levels and Akt phosphorylation. However, the addition of SR8278 effectively blocked the effects of SR9009.

### SR9009 inhibited the mitogen-activated protein kinase (MAPK) signaling pathway in lung tissue

**Fig. 8:**
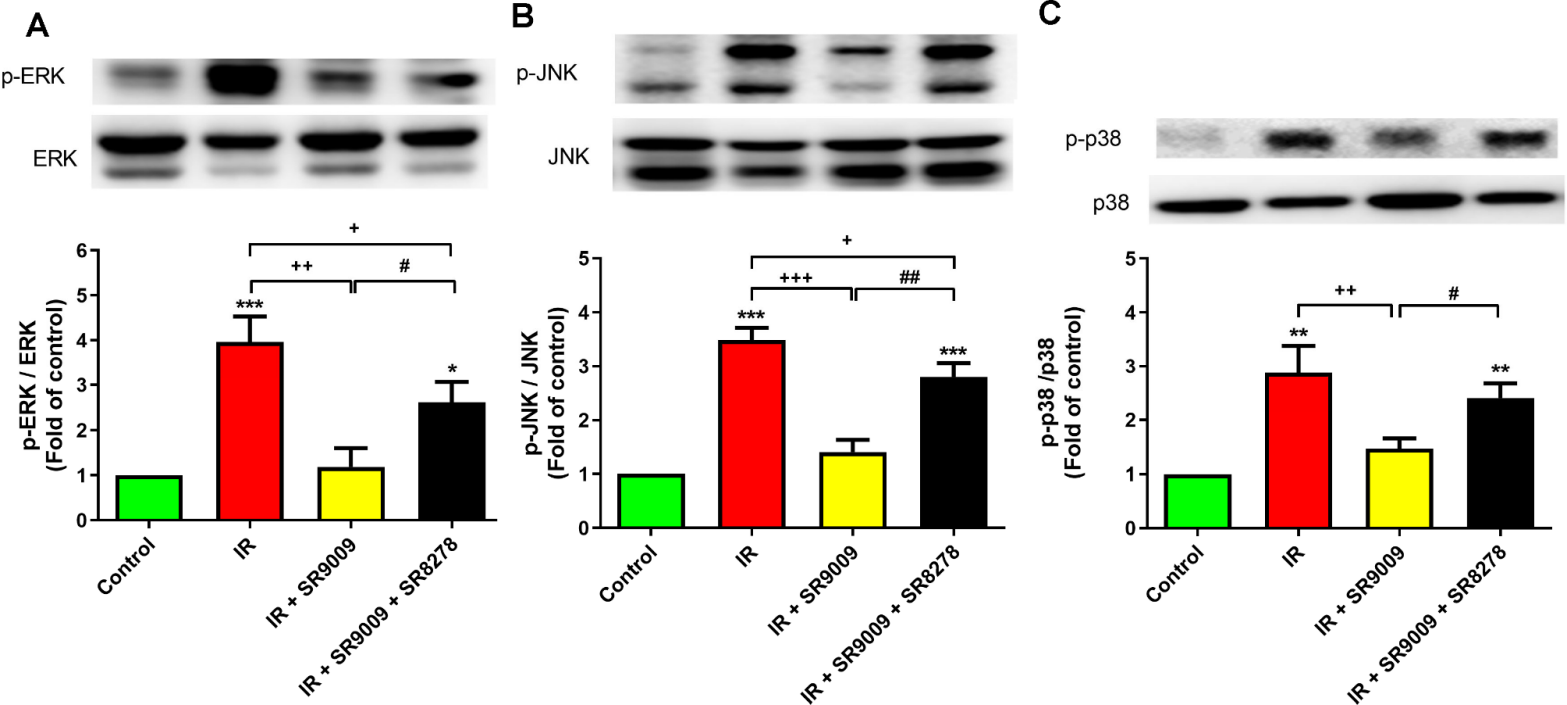
Effect of SR9009 on mitogen-activated protein kinase (MAPK) activation in lung tissues. The phosphorylation of ERK **(A)**, JNK **(B)**, and p38 **(C)** exhibited an increase in the ischemia-reperfusion (IR) group. However, the treatment with SR9009 effectively attenuated these effects. On the other hand, the addition of SR8278 negated the protective effect of SR9009. A representative blot is provided. The data are presented as mean ± SD (n = 3 per group). *p < 0.05, **p < 0.01,^***^p < 0.001, compared to control group; ^+^*p* < 0.05, ^++^*p* < 0.01, ^+++^*p* < 0.001, compared to IR group; ^#^*p* < 0.05, ^##^*p* < 0.01, compared to IR + SR9009 50 mg group

Activation of the mitogen-activated protein kinase (MAPK) pathway, including phosphorylation of ERK, JNK, and p38, was significantly increased in lung tissue due to IR. However, the administration of SR9009 successfully decreased the activation of all three MAPKs induced by IR, as shown in Fig. 8A-C. Notably, the addition of SR8278 reversed the effect of SR9009.

### Knockdown of Rev-Erbα in MLE-12 cells resulted in the suppression of SR9009-mediated protection against HR-induced injury

**Fig. 9:**
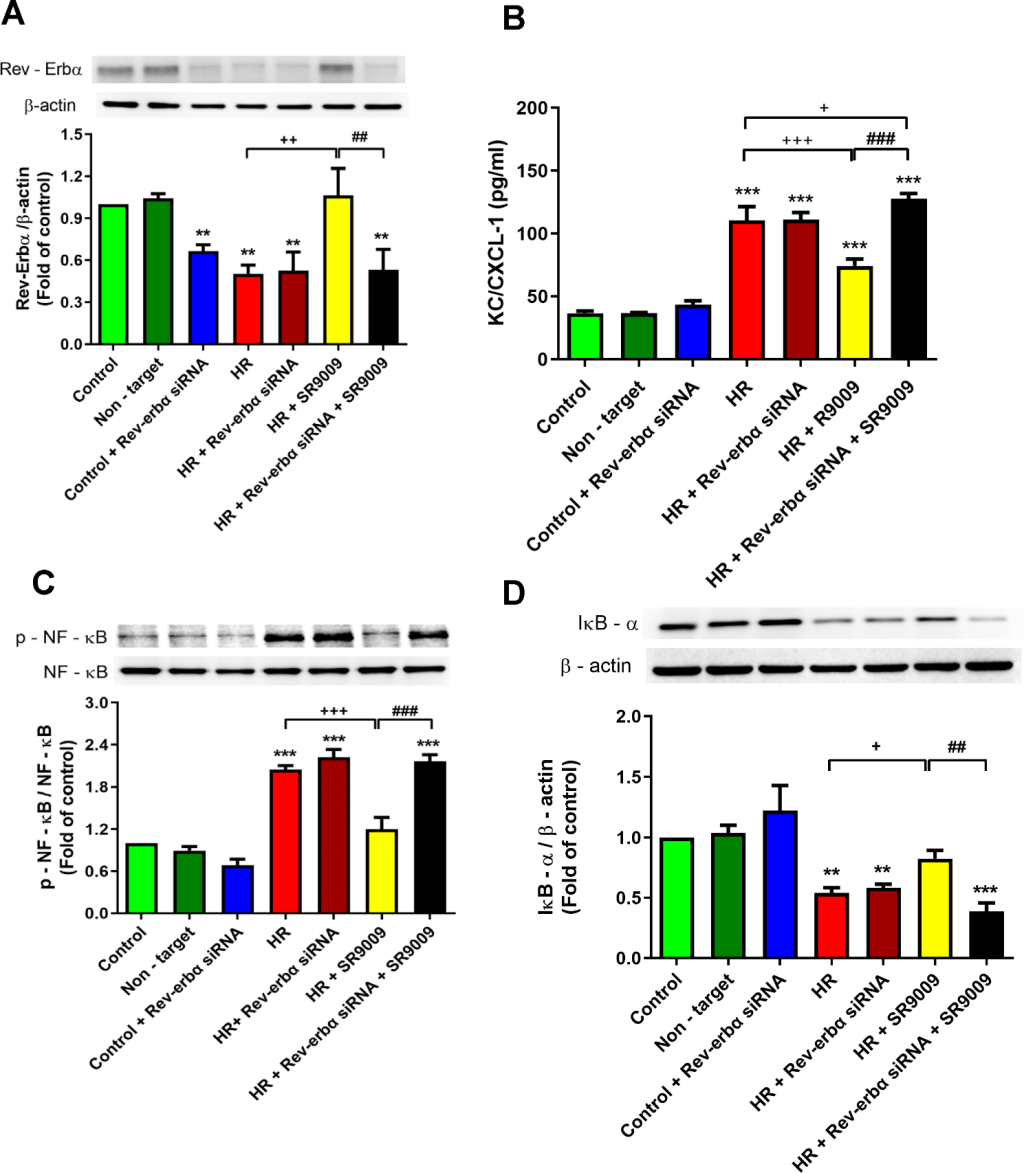
The impact of Rev-Erbα knockdown on the effects of SR9009 in MLE-12 cells during hypoxia/reoxygenation (HR) injury. **(A)** The protein expression levels of Rev-Erbα in the MLE-12 cells were assessed using Western blot analysis. Notably, SR9009 administration significantly enhanced the expression of Rev-Erbα protein in the HR group. **(B)** The production of KC/CXCL-1 in the cell culture supernatant was measured using ELISA. SR9009 effectively attenuated the increase in KC/CXCL-1 production in MLE-12 cells exposed to HR **(C)** Western blot analysis was conducted to assess the expression of IκB-α and p-NF-κB in MLE-12 cells. SR9009 notably elevated the expression level of IκB-α and reduced the p-NF-κB in MLE-12 cells subjected to 2 h of hypoxia followed by 1 h of reoxygenation. The inhibitory effect of SR9009 on NF-κB signaling and KC/CXCL-1 levels in MLE-12 cells was reversed upon transfection with Rev-Erbα siRNA. These results represent three independent experiments and are presented as means ± SD. **p < 0.01, ***p < 0.001 compared to control group; ^+^*p* < 0.05, ^++^*p* < 0.01, ^+++^*p* < 0.001, compared to HR group; ^##^*p* < 0.01, ^###^*p* < 0.001 compared to HR + SR9009 group

In MLE-12 cells from the HR group, Western blot analysis revealed a significant decrease in the expression levels of Rev-Erbα proteins compared to the control groups (*p* < 0.05; Fig. 9A). However, treatment with SR9009 resulted in a notable increase in Rev-Erbα protein expression compared to the HR group (*p* < 0.05; Fig. 9A). Furthermore, SR9009 demonstrated a reduction in the elevated levels of KC and phospho-NF-κB p65 protein induced by HR in MLE-12 cells. Additionally, SR9009 attenuated the HR-induced decrease in IκB-α protein expression. To investigate the role of Rev-Erbα protein in the beneficial effects of SR9009, siRNA was used to silence the Rev-Erbα gene in MLE-12 cells. The introduction of Rev-Erbα siRNA effectively blocked the beneficial effects of SR9009, providing further evidence of the involvement of Rev-Erbα protein (Fig. 9B-D).

## Discussion

The involvement of circadian clock dysfunction in the development and advancement of various inflammatory diseases is widely acknowledged. Nevertheless, the precise contribution of tissue-specific local circadian clocks to distinct pathophysiological processes has not been extensively investigated. In this study, we show that the pharmacologically targeting using SR9009, a Rev-Erbα agonist, successfully protected against IR-ALI in a rat model via decreasing lung edema, neutrophil infiltration, PAP, production of inflammatory cytokines, NF-κB and MAPK activation, leukocyte recruitment, and cell apoptosis. Ultimately, this reduces lung tissue damage. But the beneficial effects of SR9009 was abrogated by SR8278, a Rev-Erbα antagonist. In vitro experiments, SR9009 decreased levels of phosphorylated NF-κB p65 and KC, and increased levels of IκB-α in MLE-12 cells exposed to HR. Conversely, loss of SR9009’s target Rev-Erbα (Rev-Erbα siRNA ) abolished the protective benefits of SR9009 treatment, indicating that the effects of SR9009 are Rev-Erbα-dependent. These experiments indicate that SR9009 may have potential benefits as an adjuvant therapy for IR-ALI and that its protective mechanism may be via the Rev-Erbα signaling pathway.

Oxidative stress, caused by an imbalance between pro- and antioxidation, plays a role in the pathogenesis of ALI/ARDS [16]. Neutrophil-generated oxygen radicals can impair the integrity of the alveolar epithelial barrier, leading to increased plasma leakage and lung tissue edema. The Rev-Erbα pathway has been implicated in the modulation of oxidative stress, and it is now understood that oxidative stress can also impact Rev-Erbα pathways bidirectionally [19]. The susceptibility of Rev-Erbα transcription and circadian oscillation to oxidative stress and inflammation has been observed [19]. In contrast, Rev-Erbα enhances cellular antioxidant defense mechanisms and regulates mitochondrial energy production, thereby protecting cells against oxidative stress [20]. Our study showed that SR9009, an agonist of Rev-Erbα, reduced oxidative stress in IR-induced lung injury. These findings were supported by a reduction in H_2_O_2_ levels and lipid membrane peroxidation, as well as an increase in glutathione levels in lung tissue. SR9009 also reduced neutrophil infiltration, as indicated by a decrease in neutrophil and MPO-positive cell counts. By suppressing neutrophil-endothelium interactions, SR9009 decreased the production of proinflammatory cytokines and free radicals. These effects contributed to the attenuation of lung edema, as evidenced by lower W/D and LW/BW ratios, reduced K_f_, and decreased protein concentration in the BALF. These results are consistent with previous research showing that SR9009 can alleviate oxidative stress and reduce neutrophil infiltration in myocardial IR injury [21].

Previous investigations have implicated a complex network of inflammatory cytokines and chemokines in mediating, amplifying, and perpetuating the lung injury process [12]. It has long been thought that reducing the early inflammatory responses is a key promising strategy for intervention of ALI [22]. Our experiment showed that SR9009 significantly attenuated the increased levels of inflammatory mediators such as proinflammatory TNF-α, CINC-1 and IL-6 in the BALF after IR-ALI. Our findings were also comparable with those in previous investigations showing that SR9009 alleviated inflammatory mediator production, thereby reducing inflammation, and preventing ventilation and cigarette smoke-induced lung injury [7, 23].

The NF-κB inhibitory protein, IκB, masks the nuclear translocation signal of NF-κB, keeping it within the cytoplasm [24]. In response to inflammation, IκB undergoes phosphorylation and degradation, releasing NF-κB for nuclear translocation and activation of inflammation-associated genes such as TNF-α, CINC-1, IL-6, and CXCL-1 [13, 24]. Akt, a signaling protein, also contributes to NF-κB activation and subsequent transcriptional increase [25]. Akt-dependent events are known to play a role in ALI development [26]. Inhibition of NF-κB activation has been shown to alleviate IR-ALI severity in rats [11, 13]. SR9009, an agonist of Rev-Erbα, has been found to inhibit NF-κB signaling in various cell types and diseases. Studies have demonstrated that SR9009 suppresses LPS-induced microglial activation, hippocampal neuroinflammation, and endometrial dysfunction via the NF-κB pathway [17, 18, 27]. It also reduces cytokine production and promotes myocardial infarction survival by inhibiting the NF-κB pathway [28]. In fibroblast-like synoviocytes from rheumatoid arthritis patients, SR9009 inhibits NF-κB activation and nuclear translocation of p65 [29]. Activation of Rev-Erbα prevented colitis in mice by repressing NF-κB activity [30] Our ongoing research confirms that SR9009 inhibits the NF-κB pathway, reducing pro-inflammatory cytokine production in rat lungs exposed to IR. In an in vitro experiment using MLE-12 cells exposed to HR, SR9009 prevented IκBα degradation, inhibited NF-κB p65 phosphorylation, and reduced KC production. Conversely, a Rev-Erbα antagonist and gene knockdown had opposite effects to SR9009, both *in vivo and in vitro.*

A plethora of studies have provided compelling evidence linking apoptosis and the development of IR-ALI [31]. The anti-apoptotic properties of the Bcl-2 protein play a vital role in cellular survival and protection against IR-induced damage. In contrast, caspase-3, an effector caspase, plays a pivotal role in promoting apoptosis [32]. In the context of lung tissue, IR injury leads to a decrease in Bcl-2 levels and an increase in cleaved-caspase-3 levels, triggering apoptosis. Conversely, inhibiting apoptosis has shown promising results in ameliorating IR-ALI [12, 16, 33]. There is substantial evidence supporting a direct correlation between targeting the circadian factor Rev-Erbα pharmacologically and the downregulation of apoptosis. Previous studies conducted by other researchers have demonstrated that GSK4112, an agonist of Rev-Erbα, effectively inhibits the activation of caspase-3 in SH-SH5Y cells treated with conditioned media derived from lipopolysaccharide (LPS)-treated BV2 cells [27]. Additionally, Huang et al. have shown that SR9009 provides cardioprotection against myocardial IR injury, partly by reducing apoptosis [21]. Furthermore, Yue et al. have reported that treatment with SR9009 leads to a decrease in TUNEL-positive cells and a significant reduction in cleaved caspase-3 levels in the hippocampus after status epilepticus [34]. Consistent with these findings, our own study reveals that SR9009 effectively suppresses apoptosis in lung tissue by diminishing cleaved-caspase-3 levels and enhancing Bcl-2 expression.

The serine/threonine protein kinases known as MAPKs have been extensively studied, with p38, ERK, and JNK being the most well-known family members. These proteins actively participate in inflammatory signaling and contribute to various pathological processes associated with IR injury. Earlier studies have reported an abnormal increase in the phosphorylation of p38, ERK, and JNK in lung tissue following IR. Suppression of p38, ERK, or JNK activation has the potential to reduce IR and LPS-induced lung injury. Our investigation revealed that the administration of SR9009 effectively inhibits the effects of MAPK, thereby mitigating the extensive inflammation typically observed in IR-ALI. These findings are consistent with a study conducted by Stujanna et al., which demonstrated the ability of SR9009 to inhibit MAPK activation and improve post-myocardial infarction mortality [28]. Furthermore, Liu et al. exhibited the ability of SR9009 to suppress the phosphorylation of p38 and JNK in IL-1β-stimulated fibroblast-like synoviocytes from rheumatoid arthritis [29]. Further exploration is required to unravel the intricate mechanisms underlying the interaction between Rev-Erbα and the MAPK pathway.

Rev-Erbα, an orphan nuclear receptor, plays a crucial role in maintaining the circadian rhythm. Dysregulation of Rev-Erbα is implicated in immune and inflammatory responses triggered by environmental, inflammatory, and infectious agents [35]. Various environmental factors, including tobacco smoke, LPS, hyperoxia, allergens, bleomycin, bacterial, and viral infections, can alter the levels of Rev-Erbα in lung tissue, potentially leading to an increased DNA damage response, cellular senescence, and inflammation [35]. In our study, we observed a decline in Rev-Erbα protein levels in lung tissue following IR, while treatment with SR9009 stabilized Rev-Erbα protein expression in IR-ALI. Moreover, an in vitro experiment indicated that the protective effect of SR9009 was reversed when Rev-Erbα siRNA was used as a pretreatment. Therefore, the anti-inflammatory effect of SR9009 may be attributed to its influence on the Rev-Erbα pathway. Our results align with a previous study reporting decreased protein abundance of Rev-Erbα in cigarette smoke-induced lung inflammation, which was counteracted by SR9009 administration. Furthermore, reduced levels of Rev-Erbα have been observed in lung tissues associated with inflammatory responses in smokers, as well as in patients with chronic obstructive pulmonary disease and pulmonary fibrosis [36, 37]. It is plausible that Rev-Erbα dysfunction enhances lung inflammatory responses to environmental stressors or agents. Synthetic ligands targeting Rev-Erbα, such as SR9009, have the ability to modulate lung clock function and improve respiratory function and inflammation in individuals with asthma and COPD [36]. Consequently, Rev-Erbα initiates a series of downstream reactions that regulate host immunity and physiological processes. However, further investigation is required to uncover the precise molecular mechanisms through which Rev-Erbα affects signaling pathways.

While the involvement of Rev-Erbα in ALI pathogenesis shows promise, there are several limitations and challenges that need to be acknowledged. Firstly, it is important to note what is sex differences in the lung clock. Since we only used male rats in our experiments, our understanding of the effects of Rev-Erbα in female animals is limited. Secondly, the isolated lung model is limited in its ability to mimic the extremely complex process of ARDS in patients. Therefore, future studies should aim to verify the results in human. Thirdly, we only found links between Rev-Erbα, NF-κB or MAPK pathways. However, we did not examine the complex molecular interactions. Future experiments specifically designed to elucidate the precise mechanisms will be warranted.

## Conclusion

SR9009 effectively reduced lung edema, inflammation, oxidative stress, and apoptosis while inhibiting NF-κB and MAPK activation, alleviating IR-ALI. Yet, SR8278, the Rev-Erbα antagonist, reversed these benefits. Rev-Erbα presents a promising IR-ALI therapeutic target, demanding further investigation into its molecular intricacies and novel therapeutic avenues.

### Electronic supplementary material

Below is the link to the electronic supplementary material.

**Additional file 1**: Supplementary Figure S1. The effect of varying doses of SR9009 on pulmonary edema. Lung weight gain (**A**); vascular filtration coefficient (K_f_)(**B**); lung weight/body weight (LW/BW) (**C**); wet/dry (W/D) weight ratio (**D**); protein concentration in bronchoalveolar lavage fluid (BALF)(**E**); and pulmonary artery pressure (**F**) increased significantly in the ischemia-reperfusion (IR) group. The increase in these parameters was significantly attenuated by treatment with SR9009 in dose-dependent manner. Data are expressed as mean ± SD (n = 6 per group). *p < 0.05, **p < 0.01, ***p < 0.001 compared to control group; ^#^*p* < 0.05, ^##^*p* < 0.01, ^###^*p* < 0.001 compared to IR group

**Additional file 2**: Supplementary Fig. 2. Rev-Erbα siRNA was transfected into MLE-12 cells at different concentrations, and the expression of Rev-Erbα and β-actin (used as a loading control) was examined through immunoblotting.

## Data Availability

Data will be made available on request.

## Abbreviations

IR: Ischemia-reperfusion
ALI: Acute lung injury
ARDS: Acute respiratory distress syndrome ()
MLE-12: Mouse lung epithelial cell-12
HR: Hypoxia-reoxygenation
MAPK: Mitogen-activated protein kinase
ROS: Reactive oxygen species
BW: Body weight
LW: Lung weight
PVP: Pulmonary venous pressure
PAP: Pulmonary arterial pressure
K_f_: Capillary permeability
BALF: Bronchoalveolar lavage fluid
CINC-1: Cytokine-induced neutrophil chemoattractant-1
MDA: Malondialdehyde
JNK: Jun N-terminal kinase
ERK: Extracellular signal-regulated kinases
Akt: Protein kinase B
MPO: Myeloperoxidase
siRNA: Small interfering RNA
KC: Keratinocyte chemoattractant
NF-κB: Nuclear factor-κB
DMSO: Dimethyl sulfoxide

## References

[1] den Hengst WA, Gielis JF, Lin JY, Van Schil PE, De Windt LJ, Moens AL (2010). Lung ischemia-reperfusion injury: a molecular and clinical view on a complex pathophysiological process. Am J Physiol Heart Cir Physiol.

[2] Eltzschig HK, Eckle T (2011). Ischemia and reperfusion—from mechanism to translation. Nat Med.

[3] Fan E, Brodie D, Slutsky AS (2018). Acute respiratory distress syndrome. JAMA.

[4] Wang S, Li F, Lin Y, Wu B (2020). Targeting REV-ERBα for therapeutic purposes: promises and challenges. Theranostics.

[5] Allada R, Bass J (2021). Circadian mechanisms in Medicine. N Engl J Med.

[6] Bartman CM, Prakash YS (2020). Bringing the cellular clock into understanding lung disease: it’s time. Period! Am J Physiol Lung Cell Mol Physiol.

[7] Li H, Wang C, Hu J, Tan J (2014). A study on circadian rhythm disorder of rat lung tissue caused by mechanical ventilation induced lung injury. Int Immunopharmacol.

[8] Pariollaud M, Gibbs JE, Hopwood TW, Brown S, Begley N, Vonslow R, Poolman T, Guo B, Saer B, Jones DH (2018). Circadian clock component REV-ERBα controls homeostatic regulation of pulmonary inflammation. J Clin Invest.

[9] Yu D, Fang X, Xu Y, Xiao H, Huang T, Zhang Y, Ge Y, Li Y, Zong L, Gao J (2019). Rev-erbα can regulate the NF-κB/NALP3 pathway to modulate lipopolysaccharide-induced acute lung injury and inflammation. Int Immunopharmacol.

[10] Chu SJ, Chang DM, Wang D, Chen YH, Hsu CW, Hsu K (2002). Fructose-1,6-diphosphate attenuates acute lung injury induced by ischemia-reperfusion in rats. Crit Care Med.

[11] Wu SY, Tang SE, Ko FC, Wu GC, Huang KL, Chu SJ (2015). Valproic acid attenuates acute lung injury induced by ischemia-reperfusion in rats. Anesthesiology.

[12] Pao H-P, Liao W-I, Wu S-Y, Hung K-Y, Huang K-L, Chu S-J (2019). PG490-88, a derivative of triptolide, suppresses ischemia/reperfusion-induced lung damage by maintaining tight junction barriers and targeting multiple signaling pathways. Int Immunopharmacol.

[13] Hung KY, Wu SY, Pao HP, Liao WI, Chu SJ (2022). Acetate, a gut bacterial product, ameliorates ischemia-reperfusion induced acute lung injury in rats. Int Immunopharmacol.

[14] Liao WI, Wu SY, Wu GC, Pao HP, Tang SE, Huang KL, Chu SJ. Ac2-26, an annexin A1 peptide, attenuates Ischemia-Reperfusion-Induced Acute Lung Injury. Int J Mol Sci 2017, 18. 10.3390/ijms18081771 PMC5578160 28809781

[15] Liao WI, Wu SY, Tsai SH, Pao HP, Huang KL, Chu SJ (2021). 2-Methoxyestradiol protects against Lung Ischemia/Reperfusion Injury by Upregulating annexin A1 protein expression. Front Immunol.

[16] Tang S-E, Liao W-I, Pao H-P, Hsu C-W, Wu S-Y, Huang K-L, Chu S-J. Poloxamer 188 attenuates Ischemia-Reperfusion-Induced Lung Injury by maintaining cell membrane Integrity and inhibiting multiple signaling pathways. Front Pharmacol 2021, 12. 10.3389/fphar.2021.650573 PMC8319770 34335242

[17] Zhao W, Cui L, Huang X, Wang S, Li D, Li L, Sun Y, Du M (2019). Activation of Rev-erbα attenuates lipopolysaccharide-induced inflammatory reactions in human endometrial stroma cells via suppressing TLR4-regulated NF-κB activation. Acta Biochim Biophys Sin (Shanghai).

[18] Griffin P, Dimitry JM, Sheehan PW, Lananna BV, Guo C, Robinette ML, Hayes ME, Cedeño MR, Nadarajah CJ, Ezerskiy LA (2019). Circadian clock protein Rev-erbα regulates neuroinflammation. Proc Natl Acad Sci U S A.

[19] Yang G, Wright CJ, Hinson MD, Fernando AP, Sengupta S, Biswas C, La P, Dennery PA (2014). Oxidative stress and inflammation modulate Rev-erbα signaling in the neonatal lung and affect circadian rhythmicity. Antioxid Redox Signal.

[20] Sengupta S, Yang G, O’Donnell JC, Hinson MD, McCormack SE, Falk MJ, La P, Robinson MB, Williams ML, Yohannes MT (2016). The circadian gene Rev-erbα improves cellular bioenergetics and provides preconditioning for protection against oxidative stress. Free Radic Biol Med.

[21] Huang Q, Tian L, Zhao X, Lei S, Zhao B, Qiu Z, Xia Z-Y (2022). Rev-erbs agonist SR9009 alleviates ischemia-reperfusion injury by heightening endogenous cardioprotection at onset of type-2 diabetes in rats: down-regulating ferritinophagy/ferroptosis signaling. Biomed Pharmacother.

[22] Matthay MA, Zemans RL, Zimmerman GA, Arabi YM, Beitler JR, Mercat A, Herridge M, Randolph AG, Calfee CS. Acute respiratory distress syndrome. Nat Rev Dis Primers 2019, 5. 10.1038/s41572-019-0069-0 PMC6709677 30872586

[23] Wang Q, Sundar IK, Lucas JH, Muthumalage T, Rahman I. Molecular clock REV-ERBα regulates cigarette smoke-induced pulmonary inflammation and epithelial-mesenchymal transition. JCI Insight 2021, 6. 10.1172/jci.insight.145200 PMC8262497 34014841

[24] Liu T, Zhang L, Joo D, Sun S-C. NF-κB signaling in inflammation. Sig Transduct Target Ther 2017, 17023. 10.1038/sigtrans.2017.23 PMC5661633 29158945

[25] Romashkova JA, Makarov SS (1999). NF-kappaB is a target of AKT in anti-apoptotic PDGF signalling. Nature.

[26] Yum HK, Arcaroli J, Kupfner J, Shenkar R, Penninger JM, Sasaki T, Yang KY, Park JS, Abraham E (2001). Involvement of phosphoinositide 3-kinases in neutrophil activation and the development of acute lung injury. J Immunol.

[27] Guo DK, Zhu Y, Sun HY, Xu XY, Zhang S, Hao ZB, Wang GH, Mu CC, Ren HG (2019). Pharmacological activation of REV-ERBα represses LPS-induced microglial activation through the NF-κB pathway. Acta Pharmacol Sin.

[28] Stujanna EN, Murakoshi N, Tajiri K, Xu D, Kimura T, Qin R, Feng D, Yonebayashi S, Ogura Y, Yamagami F (2017). Rev-erb agonist improves adverse cardiac remodeling and survival in myocardial infarction through an anti-inflammatory mechanism. PLoS ONE.

[29] Liu H, Zhu Y, Gao Y, Qi D, Zhao L, Zhao L, Liu C, Tao T, Zhou C, Sun X (2020). NR1D1 modulates synovial inflammation and bone destruction in rheumatoid arthritis. Cell Death Dis.

[30] Wang S, Lin Y, Yuan X, Li F, Guo L, Wu B (2018). REV-ERBα integrates colon clock with experimental colitis through regulation of NF-κB/NLRP3 axis. Nat Commun.

[31] Tang PS, Mura M, Seth R, Liu M (2008). Acute lung injury and cell death: how many ways can cells die?. Am J Physiol Lung Cell Mol Physiol.

[32] Chen-Yoshikawa TF (2021). Ischemia–Reperfusion Injury in Lung Transplantation. Cells.

[33] Hsu H-H, Wu S-Y, Tang S-E, Wu G-C, Li M-H, Huang K-L, Chu S-J (2015). Protection against reperfusion lung injury via aborgating multiple signaling cascades by trichostatin A. Int Immunopharmacol.

[34] Yue J, He J, Wei Y, Shen K, Wu K, Yang X, Liu S, Zhang C, Yang H (2020). Decreased expression of Rev-Erbα in the epileptic foci of temporal lobe epilepsy and activation of Rev-Erbα have anti-inflammatory and neuroprotective effects in the pilocarpine model. J Neuroinflammation.

[35] Sundar IK, Yao H, Sellix MT, Rahman I (2015). Circadian molecular clock in lung pathophysiology. Am J Physiol Lung Cell Mol Physiol.

[36] Yao H, Sundar IK, Huang Y, Gerloff J, Sellix MT, Sime PJ, Rahman I (2015). Disruption of Sirtuin 1-Mediated control of circadian molecular clock and inflammation in Chronic Obstructive Pulmonary Disease. Am J Respir Cell Mol Biol.

[37] Wang Q, Sundar IK, Lucas JH, Park J-G, Nogales A, Martinez-Sobrido L, Rahman I (2023). Circadian clock molecule REV-ERBα regulates lung fibrotic progression through collagen stabilization. Nat Commun.

