# Supplementary figures and images for: Targeting Rev-Erbα to protect against ischemia-reperfusion-induced acute lung injury in rats
Source: Respir Res. 2023 Oct 12;24:247. doi: 10.1186/s12931-023-02547-7 (PMC10571317; doi:10.1186/s12931-023-02547-7)

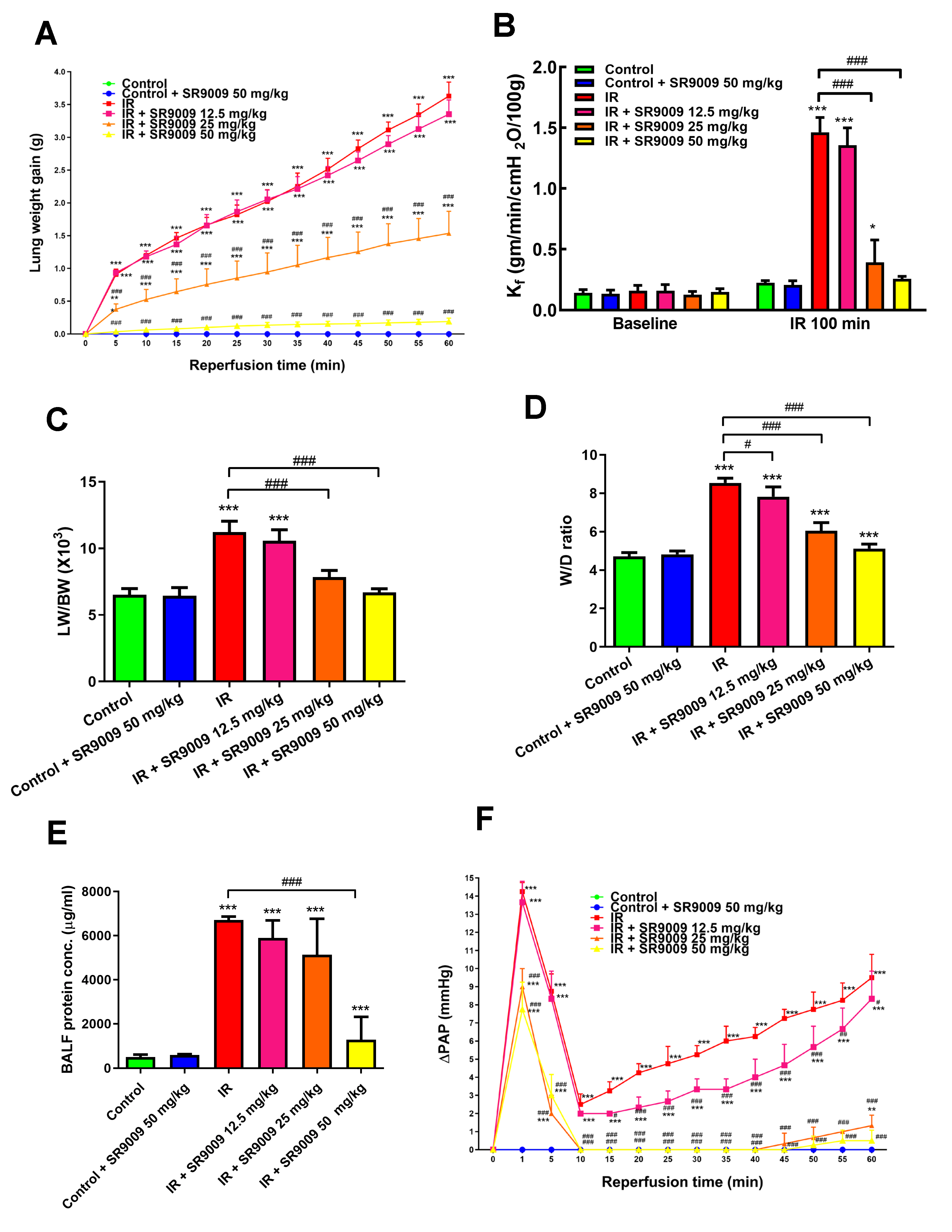

Supplement: Supplementary file 1 — Additional file 1: Supplementary Figure S1. The effect of varying doses of SR9009 on pulmonary edema. Lung weight gain (A); vascular filtration coefficient (Kf)(B); lung weight/body weight (LW/BW) (C); wet/dry (W/D) weight ratio (D); protein concentration in bronchoalveolar lavage fluid (BALF)(E); and pulmonary artery pressure (F) increased significantly in the ischemia-reperfusion (IR) group. The increase in these parameters was significantly attenuated by treatment with SR9009 in dose-dependent manner. Data are expressed as mean ± SD (n = 6 per group). *p < 0.05, **p < 0.01, ***p < 0.001 compared to control group; #p < 0.05, ##p < 0.01, ###p < 0.001 compared to IR group [file 12931_2023_2547_MOESM1_ESM.tif]

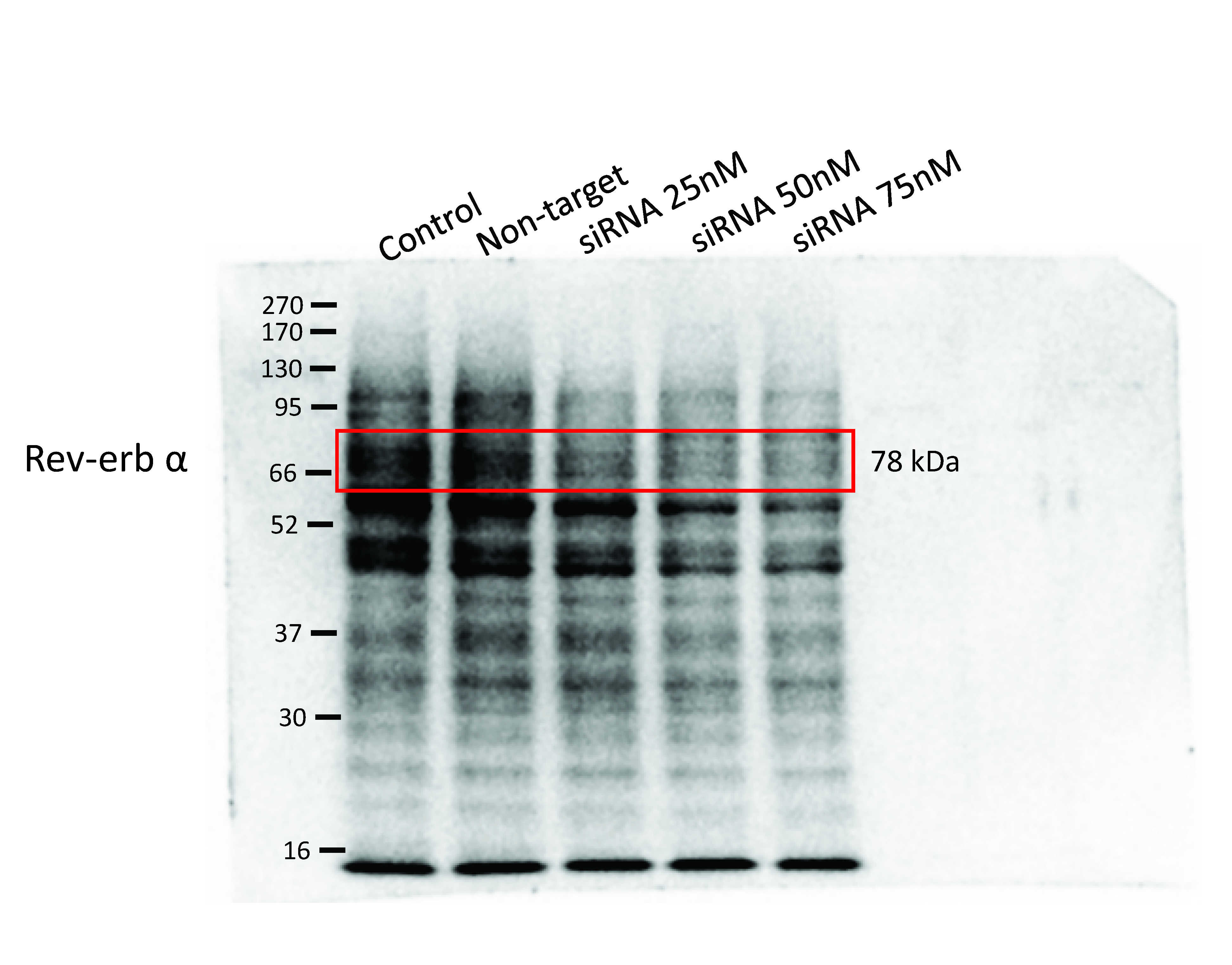

Supplement: Supplementary file 2 — Additional file 2: Supplementary Fig. 2. Rev-Erbα siRNA was transfected into MLE-12 cells at different concentrations, and the expression of Rev-Erbα and β-actin (used as a loading control) was examined through immunoblotting. [file 12931_2023_2547_MOESM2_ESM.tif]
